# Fabrication and Characterization of Polypyrrole/Multi-Walled Carbon Nanotubes Thin Films Using Thermal Evaporation

**DOI:** 10.3390/polym13224045

**Published:** 2021-11-22

**Authors:** Alaa Attar, Rima D. Alharthy, Mohammed Zwawi, Mohammed Algarni, Faisal Albatati, Mohamed Bassyouni, Mohamed Helmy Abdel-Aziz, Mohamed Shafick Zoromba, Ahmed F. Al-Hossainy

**Affiliations:** 1 Mechanical Engineering Department, King Abdulaziz University, Rabigh 21911, Saudi Arabia; loattar@kau.edu.sa (A.A.); mzwawi@kau.edu.sa (M.Z.); malgarni1@kau.edu.sa (M.A.); alalbatati@kau.edu.sa (F.A.); 2 Department of Chemistry, Science and Arts College, Rabigh Campus, King Abdulaziz University, Jeddah 21577, Saudi Arabia; iaaalharte@kau.edu.sa; 3 Department of Chemical Engineering, Faculty of Engineering, Port Said University, Port Fouad 42526, Egypt; 4 Materials Science Program, University of Science and Technology, Zewail City of Science and Technology, October Gardens, 6th of October, Giza 12578, Egypt; 5 Chemical and Materials Engineering Department, King Abdulaziz University, Rabigh 21911, Saudi Arabia; helmy2002@gmail.com (M.H.A.-A.); mzoromba@kau.edu.sa (M.S.Z.); 6 Chemical Engineering Department, Faculty of Engineering, Alexandria University, Alexandria 21544, Egypt; 7 Chemistry Department, Faculty of Science, Port-Said University, 23 December Street, Port-Said 42521, Egypt; 8 Chemistry Department, Faculty of Science, New Valley University, Al-Wadi Al-Gadid, Al-Kharga 72511, Egypt; ahmed73chem@scinv.au.edu.eg

**Keywords:** polypyrrole composites, solar cell application, multi-walled carbon nanotubes

## Abstract

Polypyrrole/multiwalled carbon nanotubes composites (PPy/MWCNTs) were produced in an acidic solution utilizing an in situ oxidative polymerization method using ferric chloride as an oxidizing agent and sodium dodecyl sulfate as a soft template. Thermal evaporation was used to fabricate thin films from polypyrrole/multiwalled carbon nanotube composites. The resulting composites were examined by different techniques to explore their morphology, structural and electrical characteristics. The surface morphology analysis revealed that polypyrrole structure is a two-dimensional film with impeded nanoparticles and the thickness of coated PPy around the MWCNTs decreases when increasing the amount of MWCNTs. XRD analysis revealed that the average crystallite size of the prepared composites is 62.26 nm. The direct energy gap for PPy is affected by a factor ranging from 2.41 eV to 1.47 eV depending on the contents of MWCNTs. The thin film’s optical properties were examined using experimental and TDDFT-DFT/DMOl^3^ simulation techniques. The optical constants and optical conductivity of the composites were calculated and correlated. The structural and optical characteristics of the simulated nanocomposites as single isolated molecules accord well with the experimental results. The nanocomposite thin films demonstrated promising results, making them a viable candidate for polymer solar cell demands. Under optimal circumstances, the constructed planar heterojunction solar cells with a 75 ± 3 nm layer of PPy/MWCNTs had a power conversion efficiency (PCE) of 6.86%.

## 1. Introduction

Conducting polymers (CPs) have recently attracted the interest of researchers due to their potential optoelectronic characteristics. Conjugated polymers with high absorption coefficients, high electrochemical activity, high conductivity, and strong chemical stability are known as CPs [1]. They have good optical and electrical qualities due to their unique physical and chemical structures and are utilized in a variety of applications such as electrochromic devices, emitting diodes, light photovoltaics, microwave shielding, electrodes for batteries, and sensors [2,3,4]. Because of its superior environmental stability and greater conductivity than many other conducting polymers, polypyrrole (PPy) is one of these conducting polymers with commercial uses. Biosensors [5,6], gas sensors [7,8], micro-actuators [9], anti-electrostatic coatings [10], polymeric batteries, electronic devices, and functional films [11], and thermoelectric materials [12] are commercial uses of PPy. PPy coatings offer high thermal stability and are well suited for application in carbon composites [13]. Converting PPy from an insulator to a conductor polymer may be done in two ways. The first method entails doping PPy with reducing reagents, which provide electrons to the polymer chains’ empty band. Negatively charged carriers are formed as a result. The second method uses oxidizing compounds as dopants, which take electrons from polymer chains and create positive charges. A P-type substance is created by these positive charges. As a result, in both fundamental research and future applications, P-type doping is favored and stressed [14]. Carbon nanotubes (CNTs) have, on the other hand, been used as fillers in a variety of applications, notably in conjunction with functional conducting polymers. Their chemical stability, electrical conductivity, and surface area are all good [15,16,17]. Polymer composites have attracted a lot of interest because they provide novel combinations with superior properties to the individual components [18,19,20,21,22].

Here, polypyrrole/multi-walled carbon nanotubes composites were created utilizing an in situ oxidative polymerization method using anhydrous ferric chloride as an oxidizing agent and SDS surfactant to regulate the shape of the resultant polypyrrole. The thermal evaporation approach was used to fabricate thin films from polypyrrole/multi-walled carbon nanotube composites.

The desired diameter in organic photovoltaic (OPV) device applications is up to 20 nm, while the usual diameter of MWNTs is between 2–10 nm and 5–100 nm [23]. Conjugated π-systems are found in both conducting polymers and carbon nanotubes. Their electrical contact is predicted to take the form of π–π stacking. CNTs (with a few exceptions) serve as electron acceptors and light is absorbed via the CNT complementary element [24,25,26,27].

Conducting polymers/CNTs composites based on polymer solar cells are of interest for developing next-generation solar cells, as CNTs are good materials for sunlight absorption and photocarrier production. Furthermore, because CNTs may create heterojunctions with conducting polymers, they can segregate carriers [28]. Furthermore, CNTs can effectively transport electrons or holes and have high PV characteristics. Although the efficiency of polymer solar cells is just a few percent, significantly lower than that of Si-based solar cells, the commercial value of polymer cells will be driven by their low-cost manufacturing and various deployment options [29]. The hybrid solar cells based on CNTs and conducting polymers are still lagging significantly behind other solar cell technologies.

In this paper, we provide a simple technique for coating multi-walled carbon nanotubes (MWCNTs) with polypyrrole to produce a one-dimensional hybrid nanostructure using an improved in situ chemical oxidative polymerization process. The inclusion of ethanol in the aqueous reaction solution and the kind of surfactant is the crucial point for regulating the polymerization rate, which increases the interfacial interaction between polypyrrole and carbon nanotubes. The optical and electrical characteristics of the material were investigated. The optical and electrical characteristics of the composites were enhanced by increasing the MWCNTs loading. The solar cell made of PPy/MWCTs composites was built. The power conversion efficiency of the cell was measured, and it was discovered that as the MWCNTs concentration grows, so does the power conversion efficiency of the cell.

## 2. Experimental

### 2.1. Raw Materials

All chemical were purchased from Sigma-Aldrich, Steinheim, Germany and were utilized as received with no additional purification. The components used in the current work are pyrrole, sodium dodecyl sulfate (SDS), ethanol, anhydrous dimethyl formaldehyde (DMF), dimethyl sulfoxide (DMSO), isopropyl alcohol, anhydrous ferric chloride, hydrochloric acid, hydrofluoric acid, chromic acid and acetic acids as well as a single crystal of (p-Si). Industrial quality multi-walled carbon nanotubes (MWCNTs) (Grafen, Ankara, Turkey) with the following parameters were used: diameter 10–40 nm, length 1.5 µm, carbon purity 90%, metal oxide 10%, and BET surface area 250–300 m^2^/g.

### 2.2. Synthesis of Polypyrrole/Multi-Walled Carbon Nanotube (MWCNT) Composites

Polypyrrole/MWCNTs composites were synthesized in accordance with previously published work [30]. At ambient temperature 3.5 g sodium dodecyl sulfate (SDS) was typically diluted in 100 mL 100% ethanol. This solution was diluted with distilled water to 400 mL using a magnetic stirrer (750 rpm) (Sigma-Aldrich, Steinheim, Germany). Separately, 0.05, 0.10, and 0.20 g of multi-walled carbon nanotubes were added to the solution. We added 4 mL of pyrrole monomer to the aforementioned dispersion, followed by 20 min in an ultrasonic homogenizer. We added 160 mL (0.5 M FeCl_3_) drop by drop to the dispersion for 1 h. Polypyrrole progressively developed during the addition phase, which occurs in the presence of MWCNTs during the polymerization process. Following the completion of the initiator addition, the resultant dispersion was kept under a magnetic stirrer for 1 h. The obtained composite was left overnight. The mixture was then filtered and rinsed with distilled water before being treated with ethanol. The resultant composite was cured for two days at 60 °C. Polypyrrole and synthesized composites were designated as PPy, [PPy/MWCNTs]-1, [PPy/MWCNTs]-2, and [PPy/MWCNTs]-3. The prepared samples are pristine polpyrrole and PPy/MWCNTs composites (PPy with different amounts: 0.05, 0.10, and 0.20 g of MWCNTs). They have been marked according to the following sequence PPy, [PPy/MWCNTs]-1, [PPy/MWCNTs]-2, and [PPy/MWCNTs]-3, respectively. Figure S1 shows the proposed synthesis scheme pyrrole oxidative polymerization reaction and combination with MWCNTs.

### 2.3. Fabrication of the Thin Films

Physical vapor deposition (PVD) was used to produce the thin films. The thin films were formed at an initial pressure of 5 × 10^−5^ mbar onto an ITO/glass substrate and/or a single crystal of wafer, with inter-digitized electrodes spaced by 75 m. A quartz crystalline micro-balance with UNIVEX 250 Leybold, two tantalum boats at any location without vacuum breakdown, and a deposition rate of 3 Å/s are used to create the film thickness of approximately 200 nm [31,32]. Figure S2 presents the steps involved in the fabrication of the films.

### 2.4. Computational Study

According to DFT calculations, simulations utilizing CASTEP and DMol3 provided findings for the efficiency of molecular structure and frequency dimensions for [PPy] and [PPy/MWCNTs] as isolated states. The applications of DMol3 and CASTEP software were computed based on free molecules, GGA functional correlations, Perdew–Burke–Ernzerh exchange, the pseudo-conserving norm, and the DNP base set [33,34]. The plane-wave cut-off energy value for the structural matrix simulation computations was 220 eV. To demonstrate the structural and spectroscopic properties of isolated [PPy] and [PPy/MWCNTs] utilizing DMol3 and CASTEP frequency calculations at the GP, including an XRD system and optical characteristics. Functional Becke’s non-local-based interchange correlation with functional B3LYP [35] and WBX97XD/6-311G were performed on doped [PPy] and [PPy/MWCNTs] as isolate state nanocomposites for optimized geometry and vibrational frequency (IR) measurements. Nanocomposite materials are evaluated for geometric parameters, vibration modes, optimal structure visualization, and energies using the GAUSSIAN 09W programmer (Carnegie Mellon University Gaussian, Inc). It has been discovered that DFT calculations are dependent on WBX97XD/6-311 G using the B3LYP method, which has resulted in numerous excellent findings for structural spectrum correlation, including some major experimental discoveries. The Gaussian Potential Approximation System (GAP) employs a range of descriptors, as well as the overall power and derivatives model, as well as the concurrent use of several unique uncertainty models, as well as the Gaussian and CASTEP models in the gas phases, to measure [PPy] and [PPy/MWCNTs].

### 2.5. Characterization

Several techniques were used to characterize the thin films, specifications are listed in Table 1.

## 3. Results and Discussion

### 3.1. Fourier Transform Infrared (FT-IR) Spectroscopy

Figure S3 shows FTIR spectra for PPY, MWCNTs, and/MWCNTs/PPy nanocomposites [12]. The 1550 and 1450 cm^−1^ bands are shown on this graph. These correspond to PPy’s stretching pulses C=C and C–N. At 1170 cm^−1^, the pyrrole ring’s vibration is generated. C–H and N–H vibrated in-plane at 1040 cm^−1^ and out-of-plane at 860 cm^−1^. The 1130 cm^−1^ band has a PPy (chloride ion doped) characteristic. The anticipated S=O expanded vibration at 1183 cm^−1^ could not be identified due to overlap with the pyrrole ring vibration at 1170 cm^−1^. The FTIR spectrum of the PPy/MWCNT composites with PPy are comparable to that of clean MWCNTs and PPy. This validates the presence of all PPy and MWCNT characteristic bands in composites. The strength of the C–H band varied dramatically as the proportion of MWCNTs increased.

### 3.2. Surface Morphology

**Figure 1:**
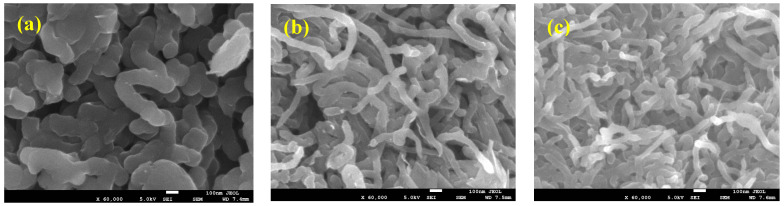
(**a**–**c**). Scanning electron microscopy (SEM) image of polypyrrole/multiwalled carbon nanotubes composites (PPy/MWCNTs) composite thin films.

The scanning electron microscope (SEM) technique was used to examine the surface morphology of the synthesized neat PPy and PPy/MWCNTs composites. Figure 1a–c showed SEM images for PPy/MWCNTs composites. From Figure 1a, it can be noticed that the obtained polypyrrole structure with impeded carbon nanotubes. The shape and size of the obtained polypyrrole usually depend on the type of used surfactant during the polymerization process. The formation of PPy sheet/films has been synthesized in the presence of SDS surfactant using FeCl_3_ as an oxidizing agent [36]. This difference depends on the synthesis conditions such as the variations in the molar ratios of the pyrrole and surfactants/oxidants, which might affect the formation of bonds between the resulting PPy chains. Figure 1b,c show SEM images of the resulting MWCNTs coated with PPy at different loadings of MWCNTs. The thickness of coated PPy around the MWCNTs decreases when increasing the amount of MWCNTs.

### 3.3. X-ray Diffraction (XRD) Analysis

**Figure 2:**
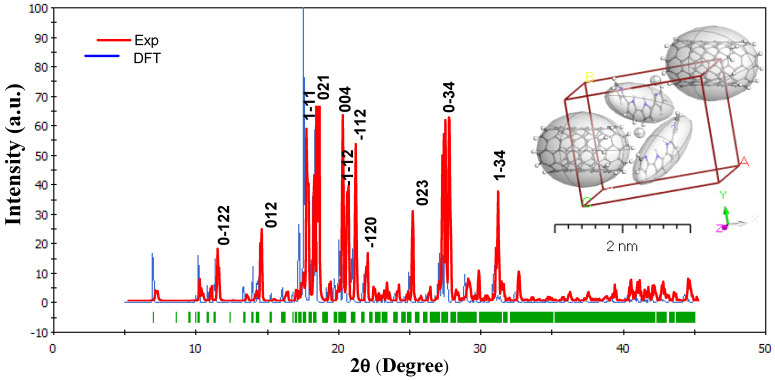
Combined between the experimental and simulated PPy/MWCNTs composites’ X-ray diffraction (XRD) patterns, inset Figure is lattice type: 3D Triclinic by using Polymorph computation method.

The XRD patterns indicate that the MWCNTs are embedded into the PPy nanocomposite. Furthermore, no significant peak shifts were observed in the XRD patterns. Figure 2 presents the pattern XRD for the experimental and simulated for PPy/MWCNTs composite. When spinning doped, the MWCNTs can be incorporated into the PPy crystal lattice. The XRD pattern obtained from the fabricated PPy/MWCNTs composites thin films. They were correlated to the isolated system matrix. The predicted crystallite size (D) and miller index (hkl) depend on the absolute values of full width at half maximum (FWHM) as given in Table 2. The data in database code amcsd 0020475 corresponds well with the interplanar distances d spacing [37]. The TDDFT-DFT and Crystal Sleuth Microsoft programs were used to designate peak lines calculated by diffraction that were close to the observed findings. The Debye–Scherrer formula was applied to assessed XRD for [PPy/MWCNTs]^0–3^ thin films, the range of 5 ≤ 2θ ≤ 45 with $1/dhkl=0.06\;{Å}^{-1}-0.74\;{Å}^{-1}$, λ=1.54 Å, $I_{2}/I_{1}=0.5$, polarization = 0.5, and Pesedo-Voigt function. From Scherer’s formula:

(1) D=0.9λ/(FWHM.cosθ)

where λ is the X-ray wavelength (1.54 Å). As presented in Table 2, for the fabricated [PPy/MWCNTs] composites thin films XRD data from the XRD pattern was used to examine factors and features such as FWHM, the crystallite size (D), hkl indices, d-spacing (d), and peak intensity. The crystallite size is $D_{av}=62.26$ nm was within the range of 32.49–144.87 nm. Polymorph calculated the theoretical X-ray diffraction models using content studio software (version 7.0) (See Figure 2). Inset Figure 2, the integrals were conducted on the Brillouin zone with 2 × 2 × 1 (Polymorph PPy/MWCNTs composites). For the corresponding experimental, a comparison was made between experimental X-rays structures and measured PXRD patterns for PPy/MWCNTs composites thin films. While both experimental and PXRD models the intensity and location of specific peaks vary only slightly, the emphasis here is mostly on their overall resemblance. Only the important comparison characteristics between the measured and the experimental data should therefore be evaluated. It is also known that instrumentation and data collection processes are only two of the many variables that can affect the experimental PXRD pattern. The simulated PPy/MWCNTs composites as isolated position in polycrystalline and provide a triclinic in the group P$\bar{1}$. For PPy/MWCNTs composites thin films, the main peaks at hkl (1$\bar{1}$1), hkl (021), hkl (004), and hkl ($\bar{1}\bar{1}$2) at 2θ values of 17.54°, 18.30°, 20.04°, and 20.38°, respectively. A full assessment demonstrating a good agreement between the calculated PXRD patterns and the experimental patterns for PPy/MWCNTs composites thin films, validating the accuracy of the fabricated material’s PXRD patterns. As presented in Figure 2, a combination of experimentally based diffraction and density functional theory calculations yields a great estimation of the atomic scale for PPy/MWCNTs composites thin films (2θ at hkl (1$\bar{1}$1).

### 3.4. Geometric Study

**Figure 3:**
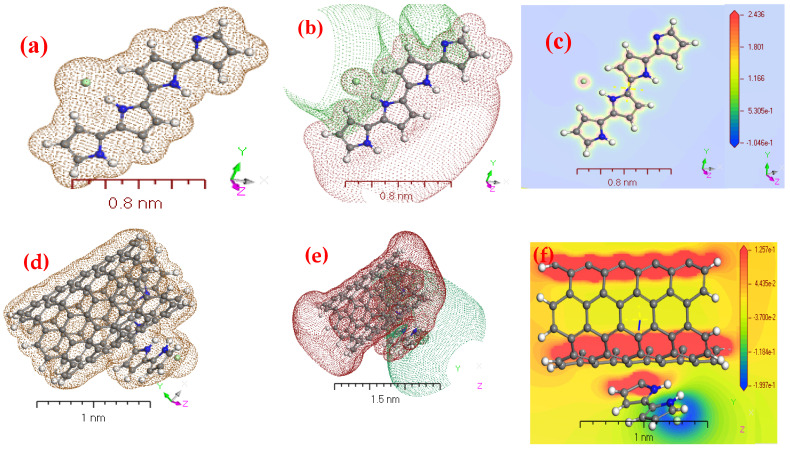
The geometry parameters in isolated state for (**a**) electron density of PPy; (**b**) potential of PPy; (**c**) electrostatic potential (MEP) of PPy; (**d**) electron density of PPy/MWCNTs composites; (**e**) potential of PPy/MWCNTs composites and (**f**) MEP of PPy/MWCNTs composites using DMOl^3^/DFT programs.

For Figure 3a–f, the similarity of physical-chemical properties of the gaseous phase of PPy/MWCNTs composites was investigated using electrostatic potential and electron density [38,39]. DFT concepts Figure 3a,d, that use electron density as an essential operator for the assessment of isolated state of PPy/MWCNTs composites gas-phase electron systems, respectively. Figure 3b,e explains potential diagrams demonstrating significant potential growth for PPy/MWCNTs composites gas-phase potential, respectively. It supports the possibility of electron transfer to PPy/MWCNTs composites in the gas phase to compute the electrostatic potential (MEP) according to its surface density. Figure 3c,f showing 3D images of the active site of the MEP during the PPy/MWCNTs composites isolated molecules, respectively. The colors blue and red demonstrate the areas advantageous for nuclear and electrical attacks. In isolated molecule phase, the potential range [P] of the PPy/MWCNTs composites matrix is 2.436 ≥ [P] ≥ −1.046 × 10^−1^ and 1.257 × 10^−1^ ≥ [P] ≥ −1.997 × 10^−1^ the color order: red < brown < blue [40,41]. The MEP diagram shows potential negative areas of positive potential for hydrogen atoms. The blue hue represents the most attraction, while the red color represents the most repulsion [42,43]. The single electronegative atom pair was aligned with negative V regions (r). The oxygen atoms of the anion of nitrogen were detected in the examined molecule in negative regions, while maximal positive regions in pyrazine2-one are local to protonated groups (–N(H)–C=C=C–N(CH)–), which may be regarded as possible nucleonic attack sites with a maximum value of +3.87 a.u. According to the following computations, the MEP map showed negative potential locations for N-atoms and positive potentials surrounding the H-atoms. These websites offer a valuable understanding of the intermolecular interactions of the chemical. The presence of intermolecular hydrogen bonding is, therefore, confirmed in Figure 3c,f.

The measured $\Delta E_{g}^{Opt}$ values were based on the highest occupied molecular orbital (HOMO) and the lowest unoccupied molecular orbital (LUMO) theory discrepancy by utilizing the DMol^3^/DFT procedure as shown in insert Figure S5.

The boundary molecular orbits in the molecular orbit (FMOs), also known as HOMO and LUMO, are important parameters in quantum chemical simulations for complex analysis (insert Figure S4). The computed energy $E_{HOMO}$, $E_{LUMO}$ and $\Delta E_{g}^{Opt}$ are presented in Table 3. The following equations were used to compute the reported values of the chemical potential, global hardness, electronegativity, global softness, global electrophilicity index, softness, and the maximum quantity of electronic charge [44].

(2) $$\mu =E_{HOMO}+E_{LUMO}/2$$

(3) $$\eta =E_{HOMO}-E_{LUMO}/2$$

(4) χ=−μ

(5) S=1/2η

(6) ω=μ2/2η

(7) σ=1/η

(8) $$\Delta N_{max}=-\mu /\eta$$

The negative values of $E_{HOMO}$ and $E_{LUMO}$ energies can be ascribed to product stability for the [PPy/MWCNTs]^0–3^ matrix. Coordination position simulation was considered for the highest magnitude molecular orbital coefficients. The electrophilicity index (t), which evaluates energy stability when the device receives an additional electronic charge, is a critical quantum chemical feature [45].

### 3.5. Optical Properties

**Figure 4:**
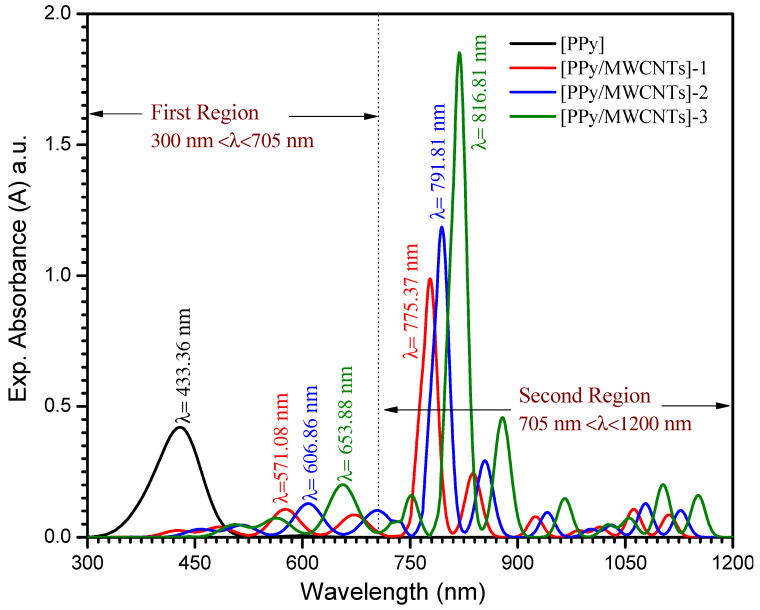
Spectra dependence of the absorption for [PPy/MWCNTs]^0–3^ thin film as experimental part.

The observation of the absorption edge in the ultraviolet field will explain crystalline/non-crystalline materials’ optical transitions and electronic band structures. Figure 4. demonstrates the PPy/MWCNTs composites thin films absorbance (a) which was computed for the normal incidence of light within a wavelength range 380 ≤ λ ≤ 1000 nm at room temperature (298 K). The relation between the absorption coefficient (α) and the transmittance (T) of a sample is α=1/d ln(1/T), where d is the thickness (d ≅ 75 ± 3 nm). The values of the absorption coefficient (α) for the [PPy/MWCNTs] composites are calculated by using the following equation:

(9) $$\alpha =1/h\upsilon \left[A{\left(h\upsilon -E_{g}\right)}^{n}\right]$$

where *A*, *E_g_*_,_ and *n* are a constant that is based on the transition probability, the band gap’s width and an index characterize the optical absorption processes in the PPy/MWCNTs composites thin films, respectively [46]. Non-linear optical effects influence a new field that changes the point, λ(nm), amplitude, or other incident fields transmission as a result of the electromagnetic fields’ relationships with different media. Non-linear optical has an important role in new applications including networking, optical links, and signal processing as the main roles of optical interference. The UV-Vis spectra, at the wavelength range (λ) 300 ≤ λ ≤ 1200 nm of the PPy/MWCNTs composites nanocomposite thin films are illustrated in Figure 4. For PPy/MWCNTs composites as-deposited thin films, in the first region, at a wavelength (λ) region 300 ≤ λ ≤ 705 nm the absorption bands are associated to n→π* electronic transition. The bands at 433, 57, 606, and 653 nm are associated with the electronic transitions from HOMO to LUMO for PPy/MWCNTs composite thin films, respectively. In the second region, at a wavelength range (λ) 705 ≤ λ ≤ 1200 nm, the absorption bands are associated with π→π* electronic transition. Likewise, the absorption bands at 775, 791, and 816 nm are associated with the electrons’ transition from valence orbital to conduct orbital for PPy/MWCNTs composites thin film as experimental parts. The UV-Vis spectra, at the wavelength range (λ) 550 ≤ λ ≤ 2900 nm of the [PPy/MWCNTs] in an isolated gaseous state as simulated part (DMOl^3^/DFT) is illustrated in Figure S3. The results of the PPy/MWCNTs in an isolated gaseous state as simulated part (DMOl3/DFT) are in good agreement with PPy/MWCNTs composites thin film which is presented in Figure 4.

**Figure 5:**
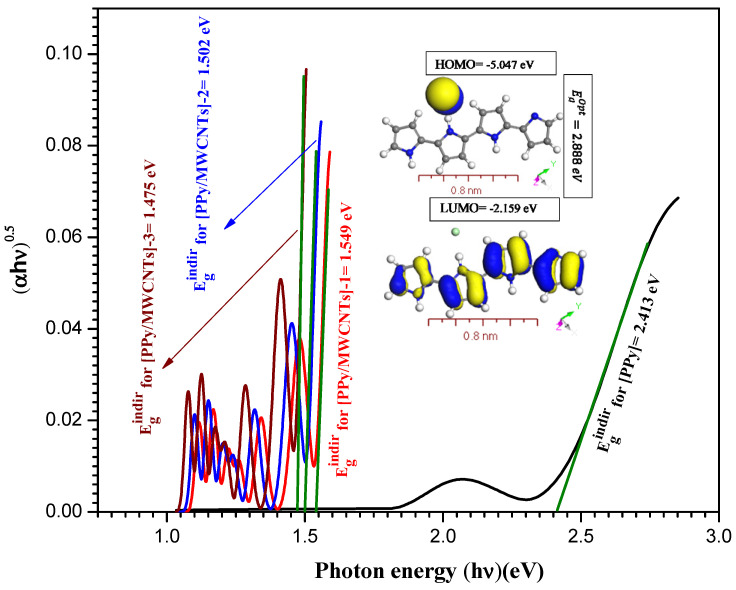
Plot indirect transitions ${\left(\alpha h\nu \right)}^{0.5}$ vs. photon energy (*hν*) eV for [PPy], Inset figure the theoretical calculation highest occupied molecular orbital (HOMO) and lowest unoccupied molecular orbital (LUMO) for PPy gas phase.

The absorption spectrum curve Abs. (λ) was used to measure the band-gap energy of the [PPy/MWCNTs] composites nanocomposite thin film. Tauc’s interaction is used to evaluate the values of ($\alpha h\nu {)}^{A}$ as

(10) $$(\alpha h\nu {)}^{A}=\epsilon_{E}^{Ind.}\left(h\nu -E_{g}\right)$$

from Abs. (λ) curve, where $\epsilon_{E}^{Ind.}$ and hν are the energy-independent constant and the incident photons energy, respectively [47]. The value A = ½ for indirect transitions ($E_{g}^{Dir})\;$ and 2 for direct allowed ($E_{g}^{Ind.}$) transitions. The expression α=Abs./d was utilized to evaluate the coefficient of absorption (α), where *d* is the thickness of the film. The $E_{g}^{Dir}$ and $E_{g}^{Ind.}$ transitions for the PPy/MWCNTs composites thin film were assessed by applying Tauc’s equation. Davis and Shilliday suggested that the direct transition ($E_{g}^{Dir})$ and indirect transition ($E_{g}^{Ind.}$) can be determined by plotting ($\alpha h\nu {)}^{2}$ and ($\alpha h\nu {)}^{0.5}$ as a function of photon energy (hν) close in the Abs. (λ) curves to zero absorption, respectively. The indirect energy values are 2.413, 1.549, 1.502, and 1.475 eV for PPy/MWCNTs composites, whereas these values of PPy/MWCNTs composites nanocomposite thin films decreased from 2.413 eV to 1.475 eV with increasing the molar ratio of MWCNTs. This decrease in $E_{g}^{Ind.}$ is because of increased disruption, which allows the electrons transformation from the valence band to the conductive band. Adding the MWCNTs decreased the bandgap as shown in Figure 5. As shown in inset Figure S4, the value of $E_{g}^{Opt}=2.888\;eV\;$ was assessed by applying the DMol^3^ process in DFT based on the discrepancy between HOMO and LUMO for free polypyrrole PPy. For Figure S4, the direct energy values are 2.648, 1.553, 1.503, and 1.48 eV for the [PPy/MWCNTs] composites, whereas the value of energy gaps for PPy/MWCNTs composites thin film decreased from 2.648 eV to 1.480 eV with increasing the molar ratio of MWCNTs. This decrease in $E_{g}^{Dir.}$ is because of increased disruption, which allows the electrons transformation from the valence band to the conductive band. As shown in inset Figure 5, the value of $E_{g}^{Opt}=1.58\;eV\;$ was assessed by applying the DMol^3^ process in DFT based on the discrepancy between HOMO and LUMO for PPy/MWCNTs composites in an isolated state. The results of the simulations by using DFT/DMOl^3^ and experimental data (Figure 4 and Figure 5) are well agreed. The equation of Tauc`s and the $E_{g}^{Opt}$ obtained can be used to evaluate electrical and energy transfer methods effectively.

**Figure 6:**
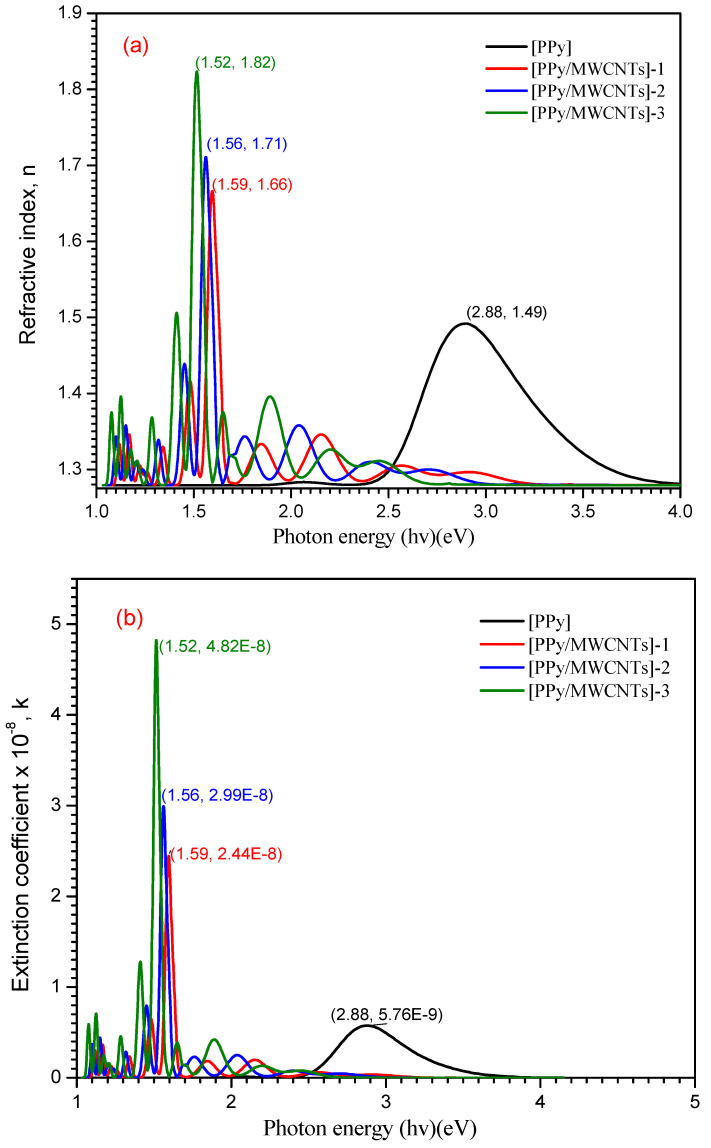

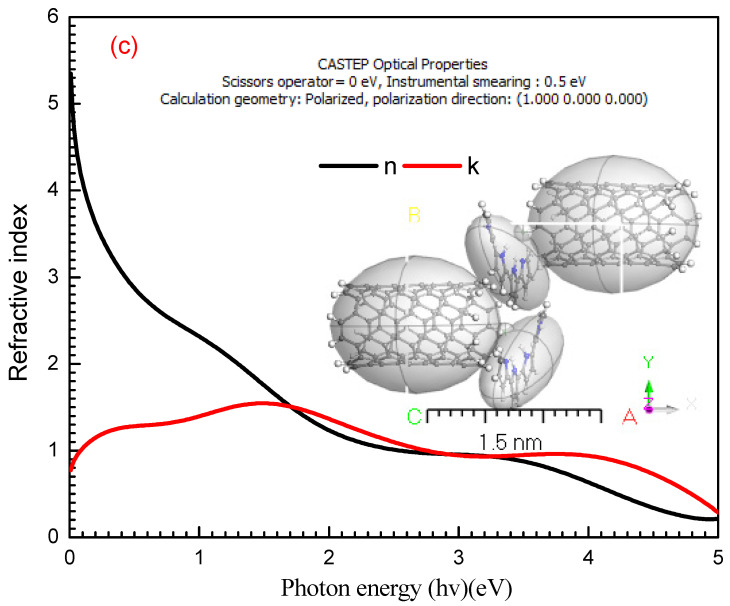
(**a**) Plot (*n*) vs. photon energy (*hν*) eV, (**b**) Plot (*k*) vs. photon energy (*hν*) eV for PPy and the different types of PPy/MWCNTs composites and (**c**) the simulated computation of (*n*) and (*k*) for PPy/MWCNTs composites as isolated molecule by using CASTEP/DFT.

The refractive index *n*(λ) is an important physical factor for a microscopic atomic interaction, which is important for polymer solar cells as well. (*n*) and (*k*) values can be calculated by applying reflectance (*R*) according to the following equations [48]:

(11) k=αλ/4π 

(12) $$n=[\left(1+\frac{R}{1}-R\right)+\sqrt{\left(\frac{4R}{{\left(1-R\right)}^{2}}\right)-K^{2}}$$

where (*n*) is the real part of the refractive index. Figure 6a–c reveals the dependence of *n*(*λ*) and *k*(*λ*)on photon energy (*hν*). As shown in Figure 6a at (*hν*) = 1.52, 1.56, 1.59, and 2.88 eV, the maximum values are 1.82, 1.71, 1.66, and 1.49 for *n*(*λ*) behavior of PPy/MWCNTs composites, respectively. From the these results, it can be concluded that the refractive index (*n*) is increased with increase the amounts of MWCNTs at the photon energy value (*hv*) = 3.938 eV, then (*n*) exhibited an increment again by increasing the wavelength until it reaches an approximate value of 1.94 at photon energy hv=1.52 eV. The absorption index *k*(*λ*) exhibited the same behavior at the same photon energy value (hv), and four peaks were observed with a maximum value of *k*(*λ*) = 4.82 × 10^−8^, 2.99 × 10^−8^, 2.44 × 10^−8^ and 2.88 × 10^−9^ which corresponds to the π→π* benzenoid rings transition for [PPy/MWCNTs] composites, respectively. From the behavior of PPy/MWCNTs composites in Figure 6b, the intensity of four peaks observed is increased with an increase in the molar ratio of CNTs, respectively. From the behavior of the simulated nanocomposite PPy/MWCNTs composites as the isolated state in Figure 6c, the CASTEP/DFT computations were used to evaluate *n*(*λ*) and *k*(*λ*) values and compared to the experimental values, simulated values are close to those achieved by DFT with the CASTEP model.

According to the single oscillator model, the dispersion curve can be described by dispersion ($E_{d}$) and oscillating energy ($E_{0}$) [49];

(13) $${\left(n^{2}-1\right)}^{-1}=\frac{E_{0}}{E_{d}}-\frac{1}{E_{0}E_{d}}{\left(hv\right)}^{2}$$

**Figure 7:**
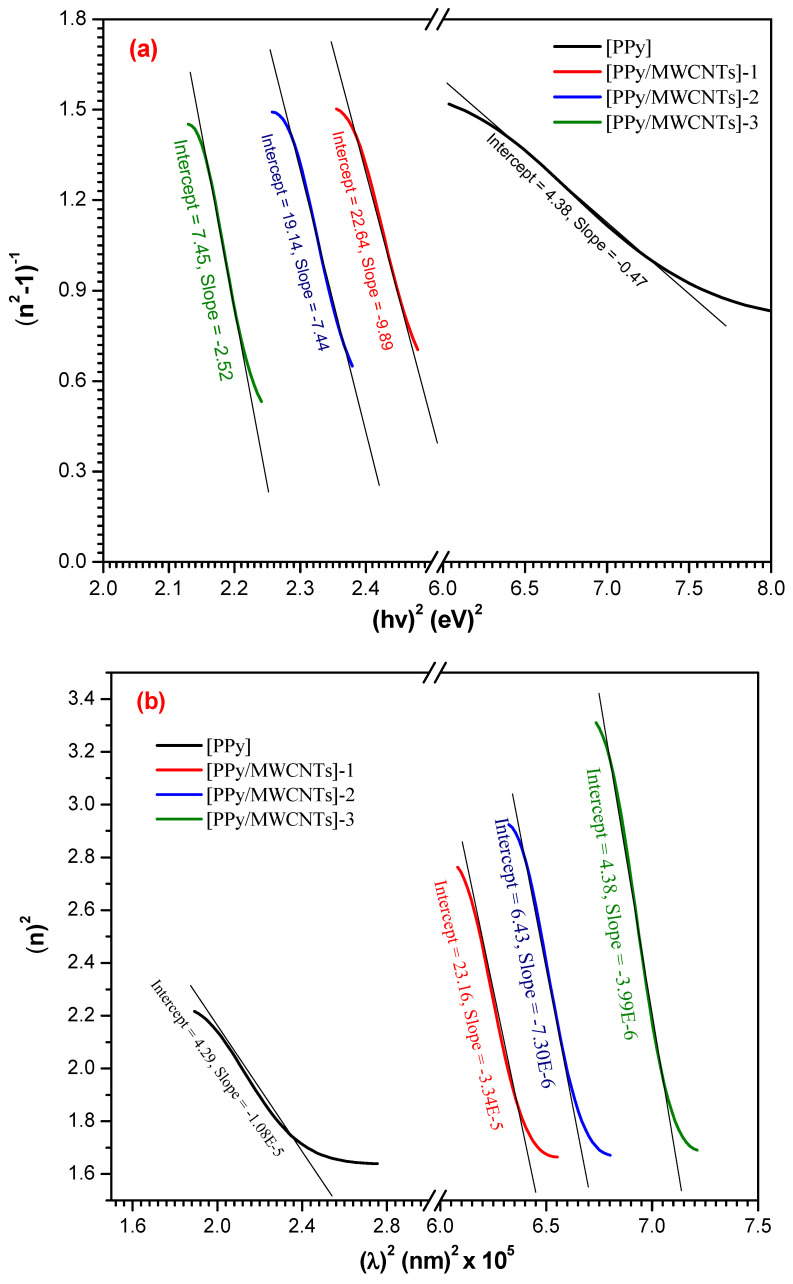

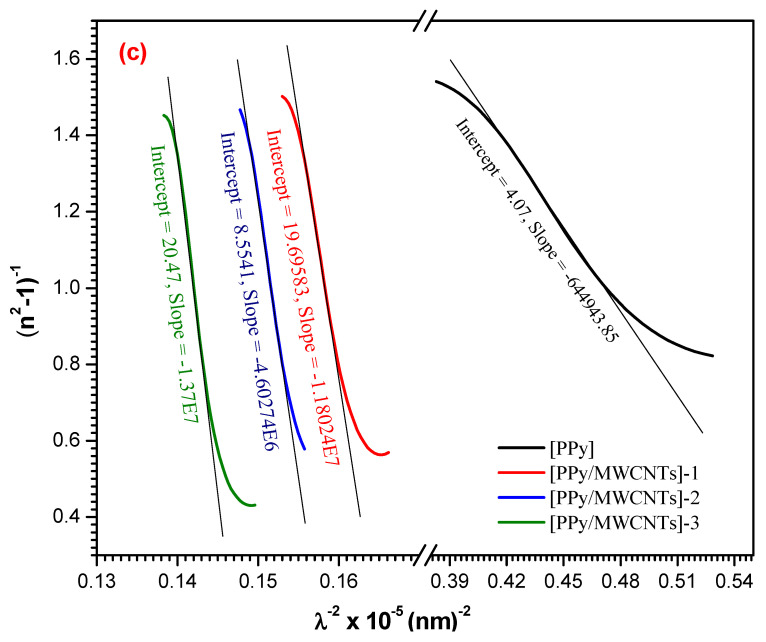
(**a**–**c**): ${\left(n^{2}-1\right)}^{-1}\;vs.\;{\left(h\nu \right)}^{2}$, $n^{2}\;\mathrm{vs}.\;\lambda^{2}$ and ${\left(n^{2}-1\right)}^{-1}\;\mathrm{vs}.\;\lambda^{-2}\;$ plots for PPy and different types of PPy/MWCNTs composites as-deposited thin films.

The ${\left(n^{2}-1\right)}^{-1}$ is characterized as (hν) (Figure 7a). Table 4 demonstrates typical dispersal parameters ($E_{d}$ and $E_{o}$), from both the slope and the linear fit of the high-frequency field. The high-frequency dielectric constant is related to the contribution produced by the electronic polarization process to [PPy/MWCNTs]^0–3^ total dielectric reaction. The oscillator strength can be computed $f=E_{0}$ and demonstrated in Table 4. The high-frequency dielectric constant can be computed as follows:

(14) $$n^{2}=\varepsilon_{\infty }-\left\{e^{2}N/4\pi^{2}\varepsilon_{0\;}c^{2}m^{*}\right\}\lambda^{2}$$

The relationship of $n^{2}$ vs. $\lambda^{2}$ is demonstrated in Figure 7b. To measure both the ratio ($N/m^{*}$) and $\varepsilon_{\infty }$, the slope and extrapolate can be employed in the resulting linear fittings. In addition, two significant periods like the one-oscillator parameters $M_{-1}$ and $M_{-3}$ depend on $E_{0}$ and $\;E_{d}$ as the following formula [50]:

(15) $$E_{0}^{2}=\frac{M_{-1}}{M_{-3}}$$

and

(16) $$E_{d}^{2}=\frac{M_{-1}^{3}}{M_{-3}}$$

To identify a single oscillator approximation to the dielectric response of the material and find the average bond strength, these moments are estimated. These optical moments are also compared to macroscopic numbers as the effective dielectric constant, effective value of electrons in the substance studied [51]. The computed $M_{-1}$ and $M_{-3}$ values of [PPy/MWCNTs]^0–3^ thin films are demonstrated in Table 4. The low $M_{-1}$ and $M_{-3}$ values provide the signal for low polarization of the examined material. The averaged wavelength (λ_0_) of the interband oscillator and the average intensity of the oscillator (*S_0_*) can be estimated by a single Sellmeier oscillator at low energy:

(17) $${\left(n^{2}-1\right)}^{-1}=\frac{1}{S_{0}\lambda_{0}^{2}}-\frac{1}{S_{0}}{\left(\lambda \right)}^{-1}$$

since λ_0_ and $S_{0}$ values can be achieved from the slope and intercept of plotting ${\left(n^{2}-1\right)}^{-1}$ against ${\left(\lambda \right)}^{-1}$ curve as demonstrated in Figure 7c and tabulated in Table 4.

The difference between ε_1_(λ) and ε_2_(λ) as a function of photon energy (hν) exposes various correlations with electrons and photons observed in PPy/MWCNTs composites as-deposited thin film. For PPy/MWCNTs composites as the deposited thin film, the n(λ) values are determined to achieve the lattice constant of a dielectric as the following formulas:

(18) $$\;\varepsilon {\left(\lambda \right)}_{1}=n{\left(\lambda \right)}_{2}-k{\left(\lambda \right)}_{2}$$

and

(19) $$\varepsilon {\left(\lambda \right)}_{2}=2nk(\left(\lambda \right)$$

where the coefficient of extinction k(λ) is gained from the relation:

(20) k(λ)=λ/4πdln(1/Abs.)

**Figure 8:**
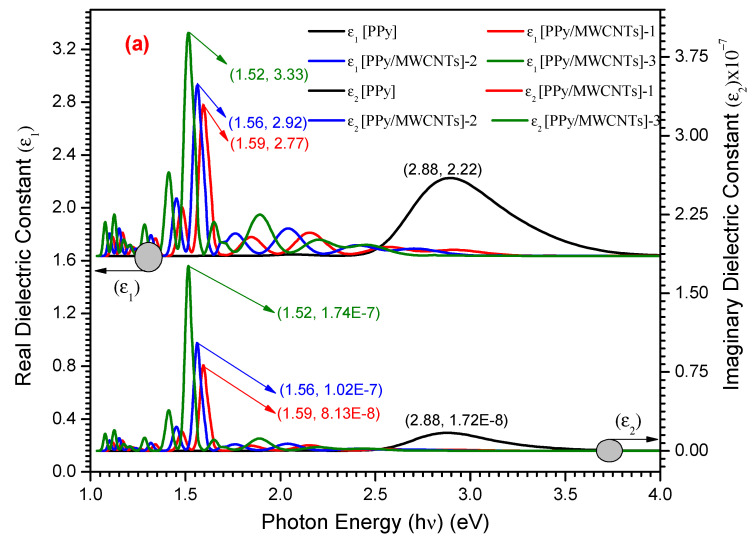

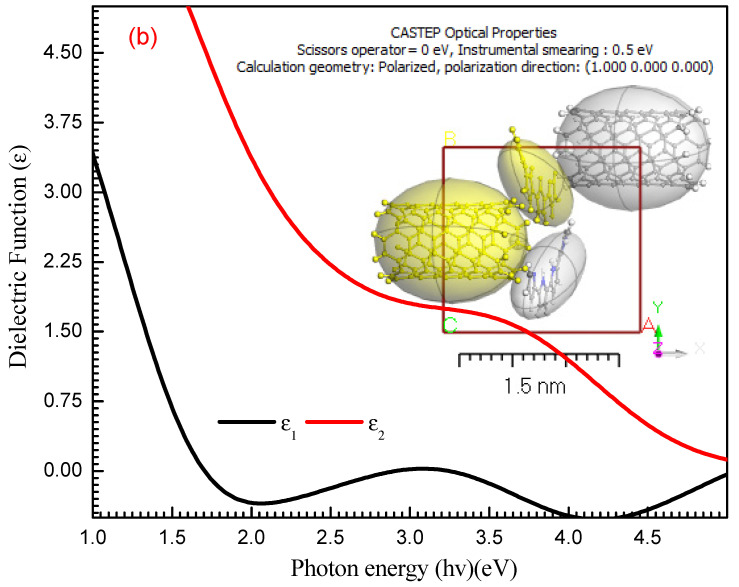
(**a**) the relation between (ε_1_ and ε_2_) vs. (hν) eV for PPy and different types of PPy/MWCNTs composites (**b**) Simulation dielectric function for PPy/MWCNTs by employing CASTEP technique.

The real ε_1_(λ) and ε_2_(λ) imaginary dielectric constants are used to illustrate the media’s response spectra to electromagnetic radiation incidents [52]. Furthermore, for both status compositions, the computed ε_1_(λ) is higher than the computed ε_2_(λ). For PPy/MWCNTs composites, in the experimental part (Figure 8), the ε_1_ (λ) show the maximum values of 2.22, 3.33, 2.92, and 2.72 for PPy/MWCNTs composites at the photon energy value of 1.52, 1.56, 1.59 and 2.88 eV, respectively. The ε_2_(λ) show the maximum values of 1.74 × 10^−7^, 1.02 × 10^−7^, 8.13 × 10^−8^ and 1.72 × 10^−8^ for PPy/MWCNTs composites at the same photon energy value of ε_1_ (λ), respectively. The high value of the computed ratio $\varepsilon_{1}\left(\lambda \right)/\varepsilon_{2}\left(\lambda \right)=1.91\times 10^{7}$ indicates that ε_1_ is predominated.

Using the CASTEP technique, the maximum value of ε_1_(λ) and ε_2_(λ) for PPy/MWCNTs composites in isolate state is ≅ 7.50 eV at photon energy (eV) ≅ 3.00 and 1.50, respectively as shown in Figure 8b. For average values ε_1_(λ) and ε_2_(λ), resulted from the experimental and simulation dimensions are found within the photon energy range values of 1–53 eV. As demonstrated in this figure, one peak was observed in the dielectric constant parts performance of PPy/MWCNTs composites. From the behavior of the simulated composite PPy/MWCNTs as an isolated state in Figure 8b, the CASTEP/DFT computations were used to evaluate ε_1_(λ) and ε_2_(λ)values and compared to the experimental values for PPy/MWCNTs composites thin films, simulated values are close to those achieved by DFT with the CASTEP model.

**Figure 9:**
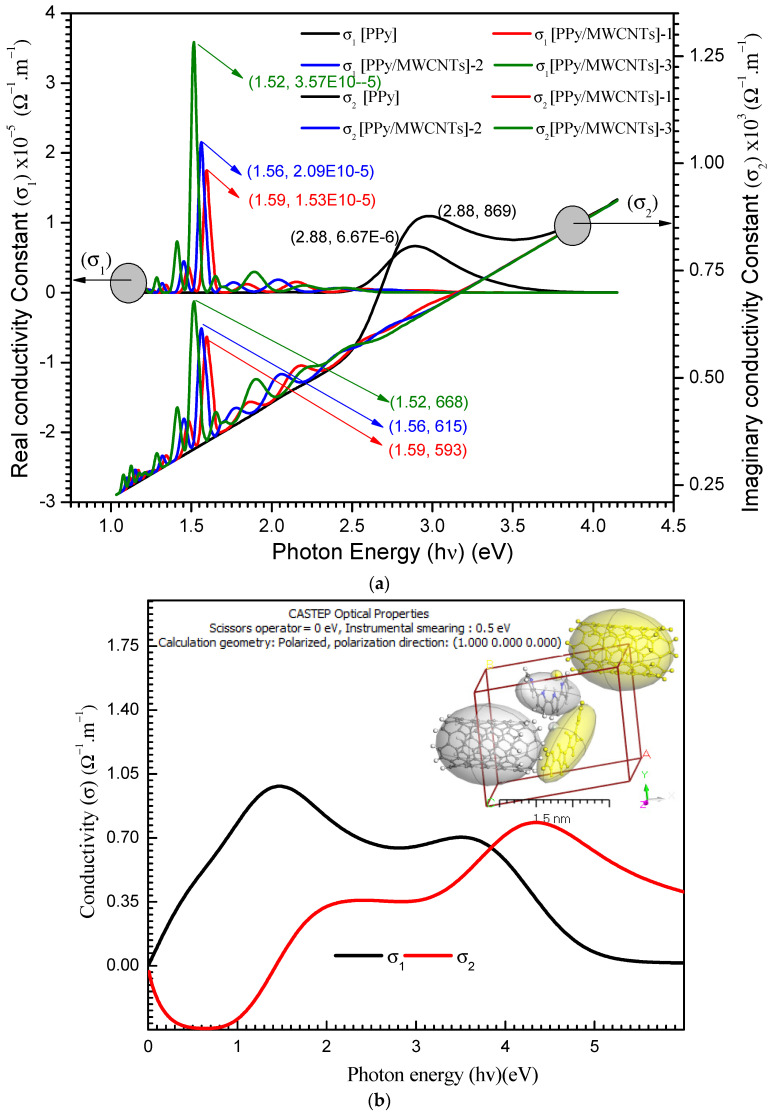
(**a**) σ_1_ & σ_2_ with (hν) eV for PPy and different types for PPy/MWCNTs composites thin films. (**b**) Simulation conductivity function for PPy/MWCNTs composites as isolated state by using CASTEP method.

To obtain the spectrum of conductivity, ε_1_(λ) and ε_2_(λ) must be combined to the consequential relationship:

(21) $$\sigma *\left(\lambda \right)=\sigma_{1}\left(\lambda \right)+\sigma_{2}\left(\lambda \right)$$

where the real part is

(22) $$\left(\sigma_{1}\left(\lambda \right)=\omega \varepsilon_{2}\left(\lambda \right)\varepsilon_{0}\right)$$

and the imaginary part is

(23) $$\left(\sigma_{2}\left(\lambda \right)=\omega \varepsilon_{1}\left(\lambda \right)\varepsilon_{0}\right)$$

fragments of the optical conductivity, (ω) and (ε_0_) are the frequency of angular and the constant of dielectric free space, respectively. The optical conductivity real and imaginary parts of PPy/MWCNTs composites thin film are shown in Figure 9a. The σ_2_ has a greater value than that of σ_1_.

For PPy/MWCNTs composites, in the experimental part (Figure 9a), the σ_1_ (λ) show the maximum values of 6.67 × 10^−6^_,_ 1.53 × 10^−5^, 2.09 × 10^−5^, and 3.57 × 10^−5^ for PPy/MWCNTs composites at the photon energy values of 1.52, 1.56, 1.59 and 2.88 eV, respectively. The σ _2_(λ) show the maximum values of 869, 593, 615 and 668 for PPy/MWCNTs composites at the same photon energy value of σ_1_ (λ), respectively. The high value of the computed ratio $\sigma_{1}\left(\lambda \right)/\sigma_{2}\left(\lambda \right)=5.34\times 10^{-3}$ indicates that $\sigma_{2}$ is predominated. The σ_1_(λ) and σ_2_(λ) values of the PPy/MWCNTs as isolate state depends on (hν) are demonstrated in Figure 9b. In the CASTEP technique, for PPy/MWCNTs, the maximum values of σ_1_(λ) and σ_2_(λ) are ≅ 0.985 Ω^−1^ m^−1^ and 0.768 Ω^−1^ m^−1^ at photon energy ≅ 1.49 and 4.40 eV, respectively. The results obtained for σ_1_(λ) and σ_2_(λ) from the experimental method and the CASTEP technique are close.

### 3.6. Electrical Properties

#### 3.6.1. The Influence of Applied Potential Difference (V) on the Current (I)

Logarithms of *V* and *I* are demonstrated in Figure S5 at different temperatures 290 ≤T(K)≤358. The nonlinear coefficient parameter (r) may come from the relationship:

(24) $$I=RV^{r}$$

where *R* is the constant, and r is the slope of these curves. There are two areas in I-V curves, the $r_{1}$ values for smaller V and $r_{2}$ for larger V with $r_{2}$ > $r_{1}$. The values of $r_{1}$ and $r_{2}$, as recorded in Table 5, confirm the non-ohmic behavior of the I-V characteristics of the investigated films. The values of the non-linear coefficient parameters for Au/[PPy/MWCNTs] composites/n-Si/Al heterojunction diode are $r_{1}\;\mathrm{and}\;r_{2}$, where the $r_{1}\;\mathrm{values}$ are less than 2, but the values of $r_{1}\;\mathrm{and}\;r_{2}$ increase with increasing MWCNTs doping ratio. In addition, raising T(K) increases $r_{1}$. On the other hand, the $r_{2}$ values are decreasing with increasing temperature. The nonlinear coefficient parameters (r) are utilized to statement the conduction mechanism in polymers [53]. We obtain ohmic behavior when r = 1. The dominant mechanism for r = 2 is trap-free space charge incomplete. Finally, if r > 2, the mechanism is defective in terms of trap charge [54]. When r is increased, traps becomes larger or deeper. Higher V values undoubtedly enhance the rate of CNT aggregation, which is regarded as undesirable and poses a problem [55]. This problem necessitates decreasing the applied potential [56].

#### 3.6.2. The Effect of Multiwalled Carbon Nanotubes Composites (MWCNTs) on Direct Current (DC) Conductivity

Aspect ratio that is high electrical percolation thresholds in MWCNT-based polymer nanocomposites (PNCs) are lower than in polymers loaded with carbon black, carbon fiber, or metals. The electrical percolation threshold is the concentration of filler at which electrical conductivity abruptly rises by many orders of magnitude [57]. The electrical characteristics of polymer nanocomposites are improved by the uniform dispersion of carbon nanotubes. The weak interfacial contact between CNTs and polymeric mixes, as well as the attraction between CNTs caused by the van der Waals force, which causes CNTs to aggregate, make achieving such a distribution difficult [58]. SEM scans indicated that an excellent dispersion of CNTs was obtained in this study. At a voltage of 20 V, DC is shown as a function of T(k). The interaction of PPy with MWCNTs boosts charge transfer inside nanocomposite films. If $\sigma_{dc}$ = 5.85 × 10^−5^ S m^−1^ [59], PPy is classified as a semiconductor material. The $\sigma_{dc}$ of PPy/MWCNTs composites are 11.99 × 10^−5^, 34.18 × 10^−5^, and 56.08 × 10^−5^ S m^−1^, respectively, which are higher than the stated value for the free polypyrrole polymer (PPy) following $E_{g}^{0}$ values (see Table 5).

**Figure 10:**
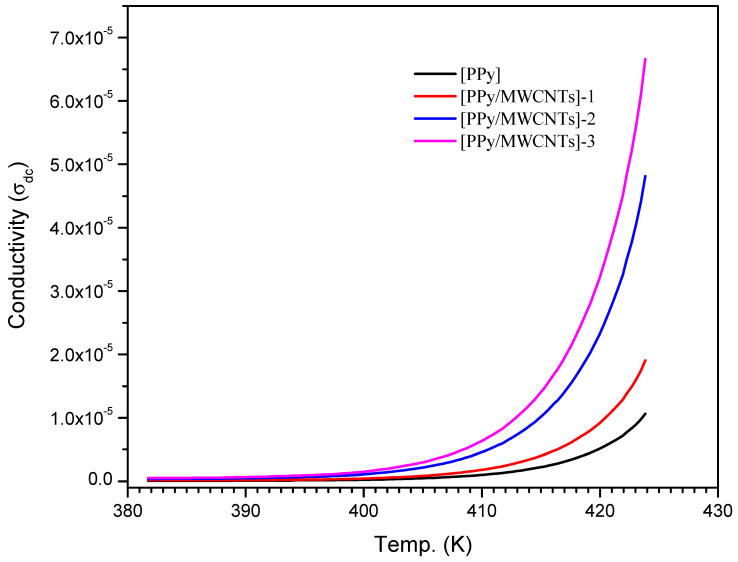
The dependence of direct current (DC) conductivity of PPy, [PPy/MWCNTs]-1, [PPy/MWCNTs]-2, and [PPy/MWCNTs]-3 on temperature (T).

$\sigma_{dc}$ reaches 1.025 × 10^−5^, 1.955 × 10^−5^, 4.765 × 10^−5,^ and 6.60 × 10^−5^ for PPy/MWCNTs composites films, nearly two orders of magnitude increase MWCNT loading was problematic due to (1) the blend’s difficulty to absorb additional MWCNTs due to PPy’s viscosity, and (2) MWCNTs’ high surface energy and inclination to agglomerate. The MWCNTs [60] can be aligned by selecting the appropriate applied electrical field (E) to decrease agglomeration and expand networks from the negative electrode to the positive electrode. The following stages are recommended for generating conducting pathways: first, the CNTs are rotated to a specific angle due to applied E, which generates a dipole moment at the MWCNT edges, aligning them in the direction of E. Second, the CNTs attract one other until they make contact, resulting in the creation of three-dimensional networks. Third, MWCNTs move to and adhere to the negative electrode. In conclusion, the electronic conductivity of CNT is the primary cause of $\sigma_{dc}$ in nanocomposite films, whereas ionic conductivity is negligible. 10^−4^–10^−5^ S m^−1^ are the values of $\sigma_{dc}$ in the semiconductor area. Raising T (i.e., semiconducting behavior) causes an increase in charge transfer, as seen in Figure 10. There are no 3D networks produced in the case of polymer and at lower CNT ratios, but the collected energy by charge carriers will activate them to leap potential barriers. Heating, on the other hand, will aid in the optimization of these routes, increasing in $\sigma_{dc}$, following increasing MWCNT content and network development.

It was reported that polypyrrole (PPy)-coated multiwalled carbon nanotubes (MWCNTs) composites were synthesized by simple, cost-effective in situ oxidative polymerization method [61]. Gas sensors were prepared in pellet form, which would be robust, cheap and reasonably sensitive to ammonia vapor sensing. The gas sensitive characteristics of composite for different MWCNTs content and over wide range of NH_3_ vapor concentration were investigated at room temperature. Also, the effect of operating temperature, humidity, long term stability was also studied. Also, the temperature dependence of DC conductivity of the PPy-MWCNT nanocomposites had been measured [62], the temperature deviation of DC conductivity was measured contained by a range 80 < T < 300 K. Resistivity decreases with the combination of MWCNTs in the PPy environment. PPy behaves as a good electron donor and MWCNTs are somewhat good electron acceptors. Therefore, there are some associations among the quinoid rings of PPy and MWCNTs which facilitate the charge transfer between the two components [63,64].

#### 3.6.3. Photovoltaic Properties of PPy/MWCNTs Composites Films

**Figure 11:**
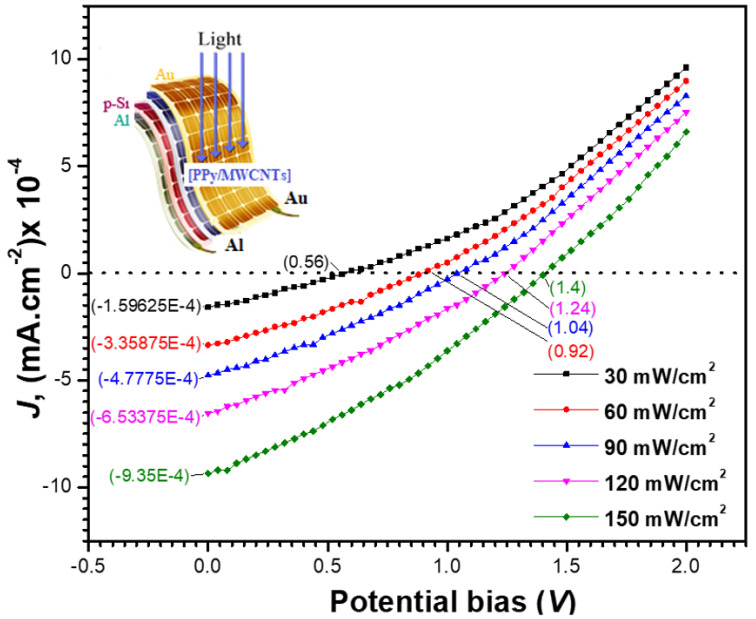
Potential bias dependence of the calculated current density under different illumination intensities. Insert is the design of the fabricated polymer solar energy.

Figure 11 shows the current density–voltage (J-V) characteristics of the Au/[PPy/MWCNTs]/n-Si/Al solar cell at various light intensities P in. In Table 6, the photovoltaic performance of the manufactured solar cell was tabulated, including the existence of current density ($J_{sc}$) at zero voltage and the existence of voltage ($V_{oc}$) at zero current density. When the intensity of the light is increased, both ($J_{sc}$) and ($V_{oc}$) values rise. The following equation is satisfied by increasing the *J_sc_* with the light intensity:

(25) $$J_{sc}=AP_{\mathrm{in}}^{\gamma }$$

where *A* is a constant. By graphing (ln *Jsc*) vs. (ln *P_in_*), the value of the exponent (*γ*) was found to be 1.28. Monomolecular and bimolecular recombination mechanisms are represented by the numbers 1.0 and 0.5 for (*γ*). The value of the exponent, on the other hand, falls between 0.5 and 1.0, indicating a continuous distribution of trapping centers. As a result, the obtained value of (*γ*) suggests that the cell under examination has a monomolecular recombination process.

The current density *J_m_* and voltage *V_m_* correlate to the power density (maximum value PM). In Table 6, the matching current density and voltage were calculated and presented. The table shows that when the light intensity increases, the values of *PM*, *J_m,_* and *V_m_* rise. The device’s fill factor (*FF*) and power conversion efficiency (*η*) were then determined using the formulae below [65]:

(26) $$FF=\frac{V_{m}J_{m}}{V_{OC}J_{SC}}\;$$

(27) $$\eta =\frac{V_{OC}J_{SC}}{P_{in}}\times FF\times 100\;$$

In Table 6, the values of *FF* and *η* were provided. It can be seen that when the light intensity is increased, the efficiency improves. At 150 mW/cm^2^, the efficiency was determined to be 6.86%.

## 4. Conclusions

Multi-walled carbon nanotubes were successfully coated by polypyrrole using in situ oxidative polymerization method. The polymerization process was carried out in an acidic medium by ferric chloride as an oxidizing agent in the presence of sodium dodecyl sulfate as a soft template. XRD data showed the experimental and simulated of PPy/MWCNTs composites had a triclinic crystal symmetry in the space group P$\bar{1}$. The crystalline size is $D_{av}=62.26$ nm, was within the range of 32.49–144.87 nm. The direct energy gap for PPy varies by a factor of 2.413 eV to 1.475 eV depending on the MWCNTs loading. The TDDFT-DFT description of geometry parameters was determined by using both DMOl3 and CASTEP techniques. The film’s I–V characteristics indicated non-ohmic behavior. They were discovered to be temperature and light-intensity-dependent. At low temperatures, the conduction mechanism in Au/n-[PPy/MWCNTs]TF/p-Si/Al was identified to be Schottky emission, but at high temperatures, the Poole–Frenkel effect was used in the [PPy/MWCNTs]TF. The current-voltage characteristics of the Au/n-[PPy/MWCNTs] thin-film/p-Si/Al heterojunction device (thickness 75 ± 3 nm) were investigated under varying illumination intensities ranging from 30 to 150 W/m^2^. As a result of the production of charge carriers, the calculated current increased dramatically with increasing light intensity. At illumination intensities of 150 W/m^2^, the resultant average value of power conversion efficiency (PCE) of an Au/n-[[PPy/MWCNTs] thin film/p-Si/Al heterojunction solar cell was computed and found to be ≅6.86%. The nanocomposite thin films demonstrated promising results, making them a viable candidate for polymer solar cell demands.

## Figures and Tables

**Table 1:** Specifications of equipment used in characterization.

Analysis	Instrumentation
SEM	SEM; (Inspect S, FEI, Holland), operated at an accelerating voltage of 3.0 kV.
Film thickness	Digital micrometer with accuracy ± 10^−3^ nm
XRD	Philips X-ray diffractometer (model X’pert) with monochromatic Cu Kα radiation operated at 40 kV.
UV	SHIMADZU UV-3101 UV–vis–NIR pc spectrophotometer.
ρ & σ	The electrical resistivity and conductivity values of the thin film were calculated by a Keithley 6517B electrometer.

**Table 2:** The computation data resulted from the application Refine Version 3.0 Software Program (Kurt Barthelme’s and Bob Downs) for PPy/MWCNTs composites.

Symmetry	Observed			Calculated		Difference			
Compound	2tdeta	d	hkl	2tdeta	d	2tdeta	d	FWHM	D_av_ ^(b)^
[PPy/MWCNTs], Triclinic	11.27	7.83	0$\bar{1}$2	11.15	7.92	−0.12	−0.08	0.19	43.23
a = 5.47(2);	14.31	6.18	012	14.41	6.14	0.10	0.04	0.26	32.49
b = 11.30(3)	17.54	5.05	1$\bar{1}$1	17.59	5.03	0.05	0.01	0.21	39.84
and c = 18.30(5) nm	18.30	4.84	021	18.25	4.85	−0.06	−0.01	0.18	46.29
α =104.7(1)°,	20.04	4.42	004	20.04	4.42	−0.003	−0.001	0.11	76.52
γ =96.4(2)°,	20.38	4.35	$\bar{1}\bar{1}$2	20.36	4.35	−0.02	−0.004	0.20	43.20
β = 89(3)°	20.56	4.24	$\bar{1}$12	20.92	4.24	−0.02	−0.004	0.11	72.58
V= 1080 (4)	20.93	4.08	$\bar{1}$20	21.75	4.08	0.02	0.004	0.16	52.12
rmse ^(a)^ = 0.000305	24.93	3.57	023	24.93	3.57	0.00	0.00	0.06	144.87
λ = 1.541838 Å	27.51	3.24	0$\bar{3}$4	27.54	3.23	0.03	0.003	0.10	81.57
machine error = 0.016	30.92	2.89	1$\bar{3}$4	30.90	2.89	−0.02	−0.002	0.17	52.12
Average								0.16	62.26

^(a)^ root mean square error. ^(b)^ nm.

**Table 3:** Geometry constant for PPy/MWCNTs composites gas phase.

Compound	$E_{HOMO}$	$E_{LUMO}$	$\Delta E_{g}^{Opt}$	χ (eV)	µ (eV)	η (eV)	S (eV)	ω (eV)	$\Delta N_{max}$	σ (eV^−1^)
PPy	−5.05	−2.16	−2.89	3.60	−3.60	1.44	0.35	4.50	2.50	0.69
[PPy/MWCNTs]	−4.20	−2.62	−1.58	3.41	−3.41	0.79	0.63	7.38	4.32	1.27

**Table 4:** Optical properties of PPy/MWCNTs composites as-deposited thin film; ($E_{g}^{Opt}$, E_U_, ($\frac{e^{2}}{\pi C^{2}}$ ) (N/m*), n_∞_, λ_∞_, $S_{0}=\left(n_{\infty }^{2}-1\right)\;⁄{\;\lambda }_{0}^{2}$, *E_d_* and *E_o_*.

**Composition**	$E_{g}^{Dir}$	$E_{g}^{Indir}$	$E_{g}^{Opt}{}^{a}$	$E_{d}$	$E_{o}$	$\varepsilon_{l}$	$\left(N/m^{*}\right)$	$\varepsilon_{\infty }$	$n_{\infty }$	$\lambda_{\infty }\;(\mathrm{nm})$	$S_{0}({\mathrm{nm}}^{-1})$
Polymer PPy	2.65	2.41	2.89	0.70	3.07	4.29	1.33 × 10^40^	1.23	1.25	197	6.31 × 10^12^
[PPy/MWCNTs]-1	1.55	1.55	1.58	0.07	1.51	4.33	4.10 × 10^40^	1.04	1.05	215	1.09 × 10^12^
[PPy/MWCNTs]-2	1.50	1.50		0.08	1.60	4.38	4.89 × 10^39^	1.05	1.12	251	1.86 × 10^12^
[PPy/MWCNTs]-3	1.48	1.48		0.23	1.72	6.43	8.95 × 10^39^	1.13	1.05	181	1.5 × 10^12^

^a^ by using simulation DMol^3^ method in DFT.

**Table 5:** The values of the nonlinear coefficient parameters ($r_{1}\;\mathrm{and}\;r_{2}$) for Au/[PPy/MWCNTs] composites/n-Si/Al heterojunction diode.

**Temp. (K)**	**Pure PPy**		PPy/MWCNTs-1		PPy/MWCNTs-2		PPy/MWCNTs-3		Activation Energy
	$r_{1}\;$	$r_{2}\;$	$r_{1}\;$	$r_{2}$	$r_{1}$	$r_{2}$	$r_{1}$	$r_{2}$	$E_{g}^{0}\;(\mathrm{eV})$
290	1.16	1.93	1.25	2.15	1.31	2.31	1.83	2.37	$E_{g}^{0}\;\left[PPy\right]=$2.78
307	1.19	1.72	1.36	1.73	1.45	2.02	1.66	2.18	$E_{g}^{0}\;\left[PPy/CNTs\right]-1=1.58$
324	1.22	1.53	1.48	1.82	1.37	1.93	1.90	2.02	$E_{g}^{0}\;\left[PPy/CNTs\right]-2=1.53$
341	1.17	1.45	1.40	1.48	1.60	1.74	1.71	2.22	$E_{g}^{0}\;\left[PPy/CNTs\right]-3=1.50$
358	1.18	1.40	1.42	1.34	1.57	1.45	1.59	2.20	

**Table 6:** I–V parameters and photovoltaic parameters of the Au/[PPy/MWCNTs] composites/n-Si/Al solar cell under different illumination intensities $P_{in}$.

Int. ^a^	*V_m_* ^b^	*J_m_* ^c^	*V_oc_* ^b^	*J_sc_* ^d^	*Power*	*FF*	*η (PCE)*
30	0.17	1.48 × 10^−5^	0.56	1.60 × 10^−4^	2.46 × 10^−6^	0.03	1.31
60	0.23	5.08 × 10^−5^	0.92	3.36 × 10^−4^	1.15 × 10^−5^	0.04	3.06
90	0.27	1.08 × 10^−4^	1.04	4.78 × 10^−4^	2.89 × 10^−5^	0.06	5.15
120	0.27	1.65 × 10^−4^	1.24	6.53 × 10^−4^	4.51 × 10^−5^	0.06	6.01
150	0.29	2.22 × 10^−4^	1.4	9.35 × 10^−4^	6.43 × 10^−5^	0.05	6.86

a = (mW cm^−2^), b = Volt, c = (mA cm^−2^), and d = (mA cm^−2^).

## Data Availability

The data presented in this study are available on request from the corresponding author.

## Supplementary Materials

The following are available online at https://www.mdpi.com/article/10.3390/polym13224045/s1, Figure S1: Fabrication and characterization of the thin films [PPy/MWCNTs] by using Physical Vapor Deposition (PVD); Figure S2: A scheme of pyrrole oxidative polymerization reaction and combination with MWCNTs to the formation of PPy/MWCNTs composites; Figure S3: FTIR spectra of the MWCNTs/PPy nanocomposites. Spectra of the pure MWCNTs and PPy are also shown.; Figure S4: Plot direct (αhν)2 vs. photon energy (hν) eV for [PPy/MWCNTs] composites thin film, theoretical calculation HOMO and LUMO for [PPy/MWCNTs] composites gas phase.; Figure S5: I–V characteristics curves and Log J for (a) Au/[PPy]/n-Si/Al; (b) Au/[PPy/MWCNTs]-1 composites/n-Si/Al; (c) Au/[PPy/MWCNTs]-2 composites/n-Si/Al and (d) Au/[PPy/MWCNTs]-3 composites/n-Si/Al heterojunction diode.
