# Supplementary material for: Fabrication and Characterization of Polypyrrole/Multi-Walled Carbon Nanotubes Thin Films Using Thermal Evaporation
Source: Polymers (Basel). 2021 Nov 22;13(22):4045. doi: 10.3390/polym13224045 (PMC8620608; doi:10.3390/polym13224045)
Supplement: Supplementary file 1 [file polymers-13-04045-s001.zip › polymers-1461044-supplementary.pdf]

Fabrication and characterization of polypyrrole/multi-walled carbon nanotubes thin films using thermal evaporation

Alaa Attar [1], Rima D. Alharthy [2], Mohammed Zwawi [1], Mohammed Algarni [1], Faisal Abatati [1], Mohamed Bassyouni [3,4,*], Mohamed Helmy Abdel-Aziz [5,6], Mohamed Shafick Zoromba [5,7] and Ahmed F. Al-Hossainy [8]

1 Mechanical Engineering Department, King Abdulaziz University, Rabigh 21911, Saudi Arabia
2 Department of Chemistry, Science and Arts College, Rabigh Campus, King Abdulaziz University, Jeddah, 21577, Saudi Arabia
3 Department of Chemical Engineering, Faculty of Engineering, Port Said University, Port Fouad 42526, Egypt
4 Materials Science Program, University of Science and Technology, Zewail City of Science and Technology, October Gardens, 6th of October, Giza 12578, Egypt
5 Chemical and Materials Engineering Department, King Abdulaziz University, Rabigh 21911, Saudi Arabia
6 Chemical Engineering Department, Faculty of Engineering, Alexandria University, Alexandria 21544, Egypt
7 Chemistry Department, Faculty of Science, 23 December Street, 42521, Port-Said University, Port-Said, Egypt
8 Chemistry Department, Faculty of Science, New Valley University, 72511 Al-Wadi Al-Gadid, Al-Kharga, Egypt
* Correspondence: m.bassyouni@psu.edu.eng

2. Experimental

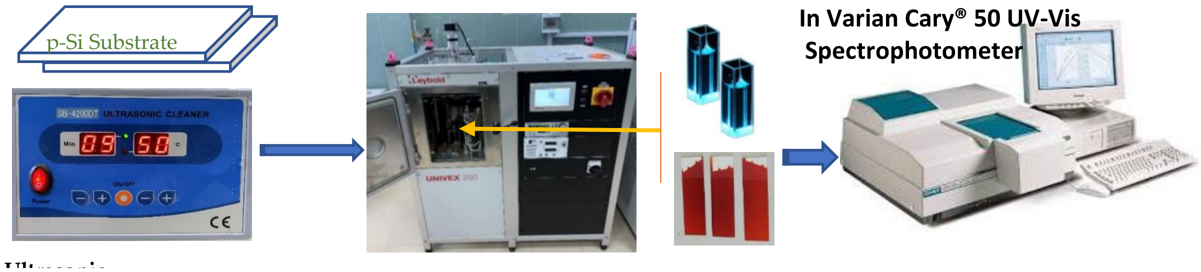

Figure S1. Fabrication and characterization of the thin films [PPy/MWCNTs] by using Physical Vapor Deposition (PVD).

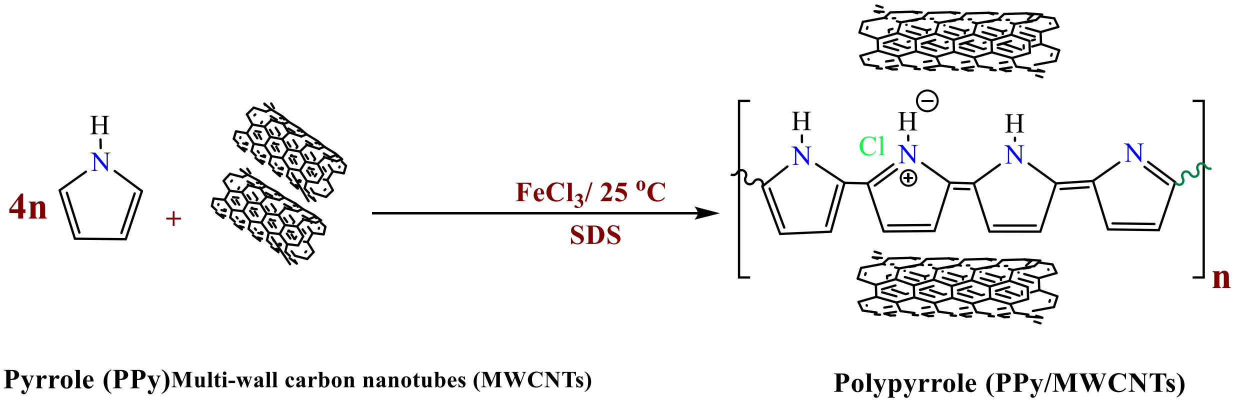

Figure S2. A scheme of pyrrole oxidative polymerization reaction and combination with MWCNTs to the formation of PPy/MWCNTs composites

3.1. FT-IR spectroscopy

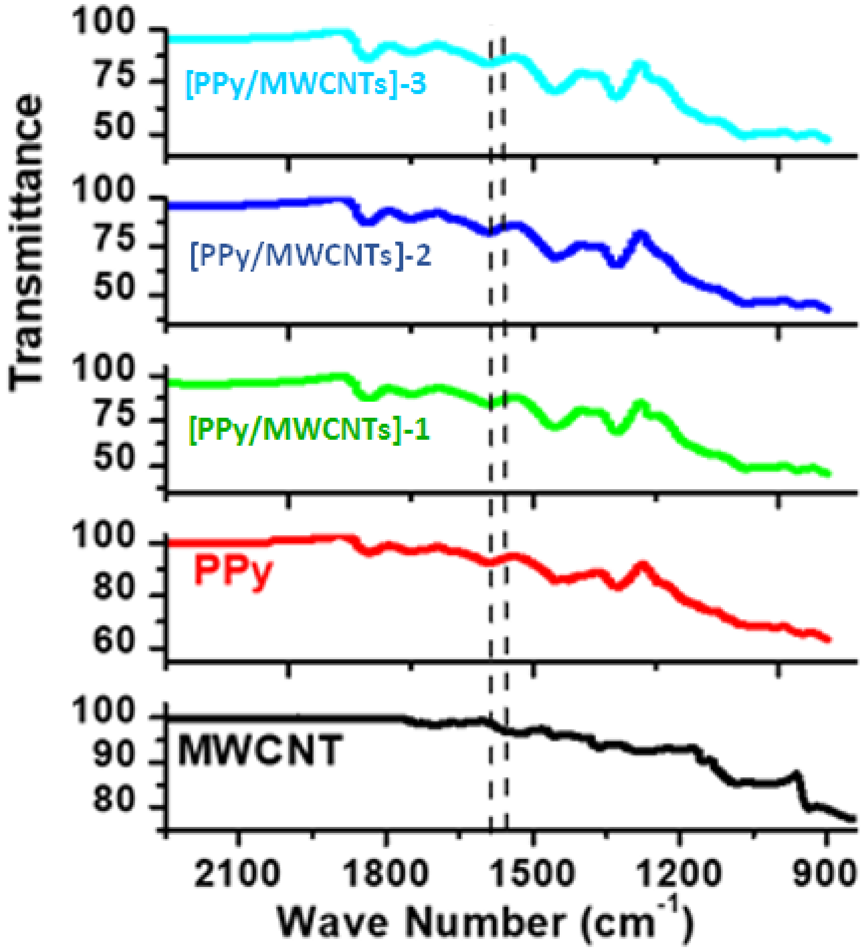

Figure S3. FTIR spectra of the MWCNTs/PPy nanocomposites. Spectra of the pure MWCNTs and PPy are also shown.

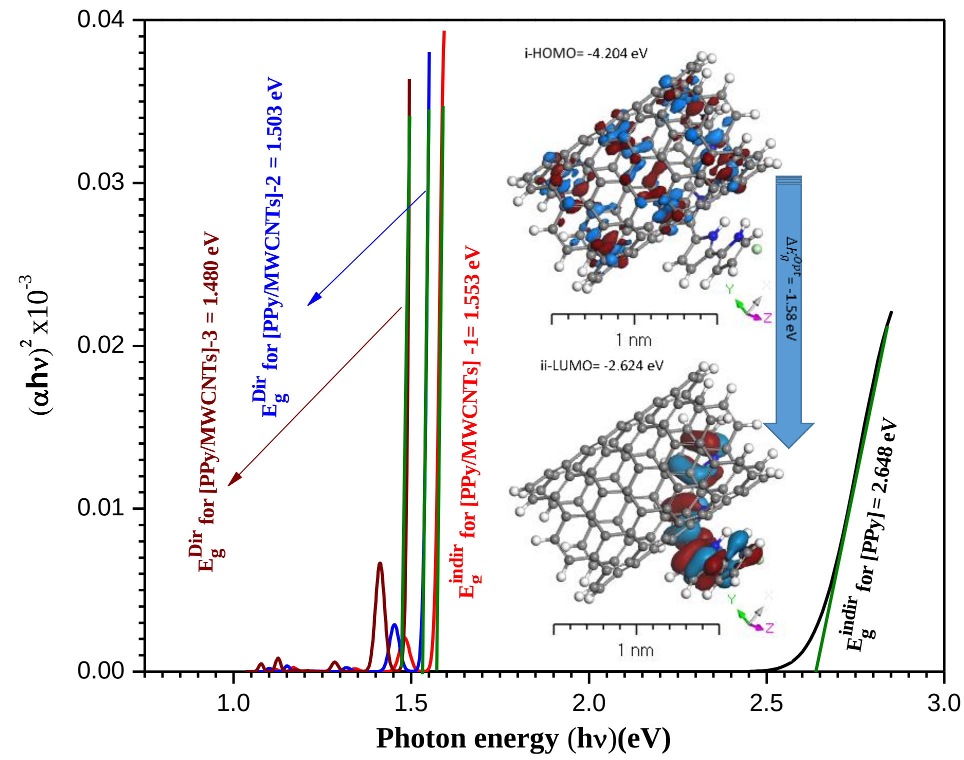

Figure S4. Plot direct $(\alpha h\nu)^2$ vs photon energy (hν) eV for [PPy/MWCNTs] composites thin film, theoretical calculation HOMO and LUMO for [PPy/MWCNTs] composites gas phase.

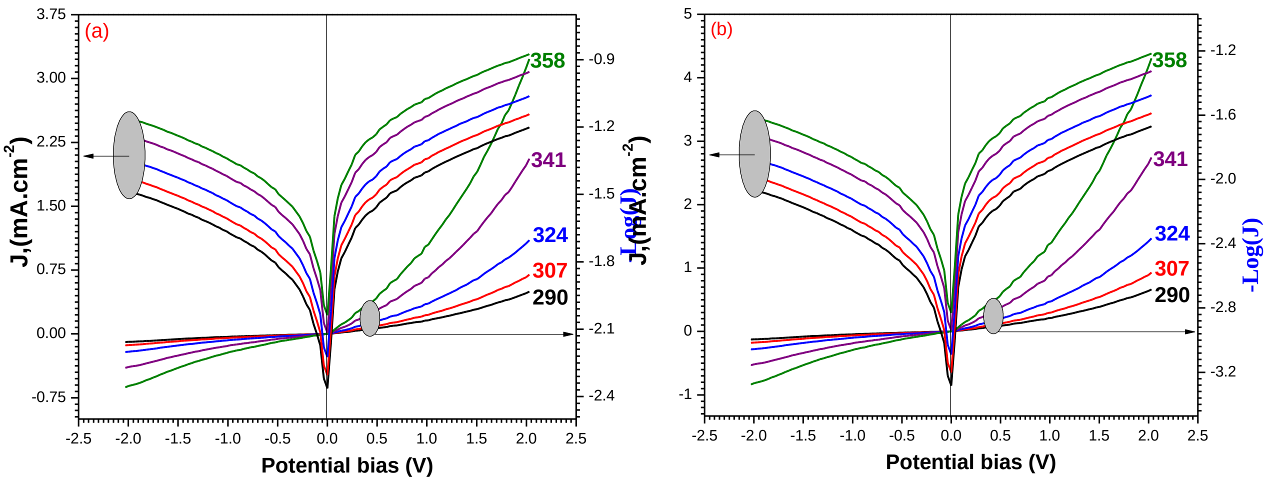

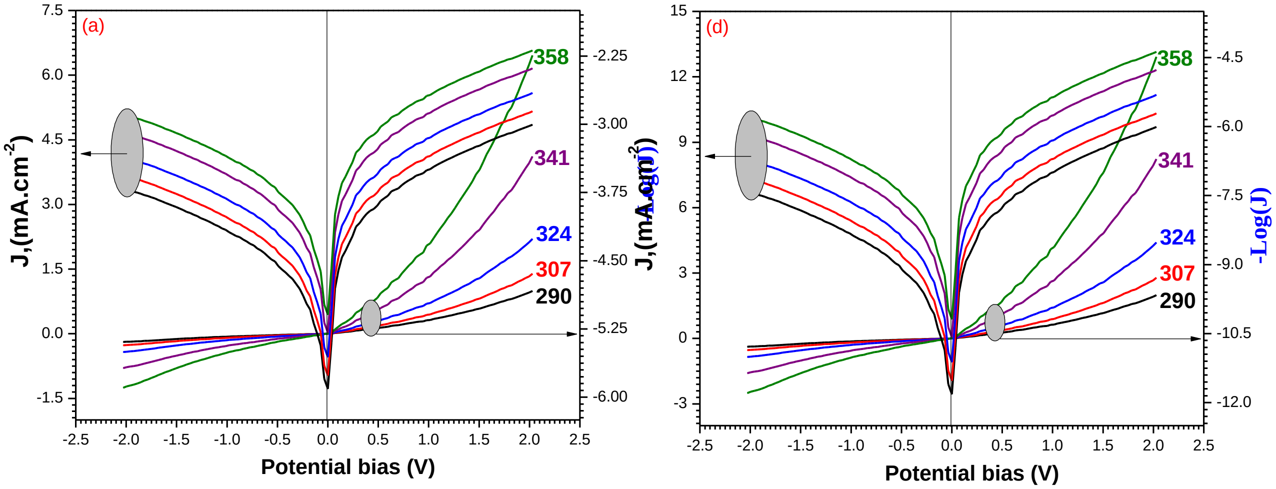

Figure S5. I -V characteristics curves and Log J for a) Au/[PPy]/n-Si/Al; b) Au/[PPy/MWCNTs]-1 composites/n-Si/Al; c) Au/[PPy/MWCNTs]-2 composites/n-Si/Al and d) Au/[PPy/MWCNTs]-3 composites/n-Si/Al heterojunction diode
